# Early Surgical Outcome of Hepatoblastoma in Children Receiving Chemotherapy After Hepatic Resection

**DOI:** 10.7759/cureus.80334

**Published:** 2025-03-10

**Authors:** Abdullah All Mahmud, Zahid Hossain, Mahfuz Alam Khan, A M Shahinoor, Umme Habiba Dilshad Munmun, Meherun Khan Methila, Syeda Sushmita Zafar, Tanjirul Islam

**Affiliations:** 1 Department of Pediatric Surgery, Shaheed Suhrawardy Medical College and Hospital, Dhaka, BGD; 2 Department of Pediatric Surgery, Bangabandhu Sheikh Mujib Medical University, Dhaka, BGD; 3 Department of Pediatric Surgery, Mymensingh Medical College Hospital, Mymensingh, BGD; 4 Department of Pediatric Surgery, Rangpur Medical College and Hospital, Rangpur, BGD; 5 Department of Community Medicine, Saic College of Medical Science and Technology, Dhaka, BGD

**Keywords:** hepatic echotexture, hepatic regeneration, hepatic resection, hepatoblastoma, liver function tests (lfts), neoadjuvant chemotherapy

## Abstract

Objective: The aim of this study was to evaluate surgical outcomes in children with hepatoblastoma who underwent hepatic resection after receiving neoadjuvant chemotherapy.

Methodology: A prospective observational longitudinal study was conducted from February 2019 to July 2020 in the Department of Pediatric Surgery at Bangabandhu Sheikh Mujib Medical University, Bangladesh. A total of 13 children diagnosed with hepatoblastoma and classified as PRETEXT (Pre-Treatment Extent of Disease) stages I to III were included. Detailed medical histories were recorded, and diagnoses were confirmed through histopathological analysis. Preoperative evaluations included liver function tests (LFTs), serum alpha-fetoprotein (AFP) levels, and imaging for tumor staging and liver volume. Postoperative assessments were conducted at one, three, and six months to monitor changes in serum AFP levels, LFTs, liver volume, and hepatic echotexture. The type of hepatic resection performed and any complications encountered were also documented.

Results: Among the 13 patients, the majority were male, with a male-to-female ratio of 12:1. The average age at diagnosis was 4.44 years, with most patients under three years old. Pathological analysis revealed epithelial tumors in 38.45% of cases, fetal-type tumors in 46.15%, and mixed tumors in 15.4%. PRETEXT stage III was the most common (53.85%), and 61.54% of patients underwent major hepatic resections. Postoperative serum AFP levels showed a significant decline, reflecting successful tumor resection and improvements in LFTs. Improvements in LFTs, including key enzymes like ALT and AST, were observed. A marked increase in hepatic regeneration was observed within six months, with no local recurrences recorded.

Conclusions: This study highlights the effectiveness of combining hepatic resection with neoadjuvant chemotherapy in treating pediatric hepatoblastoma. A significant decline in serum AFP levels after surgery reflects the success of tumor removal, while improvements in LFTs underscore the recovery of hepatic health. Furthermore, the observed increase in hepatic regeneration within six months demonstrates the liver’s remarkable ability to recover and sustain long-term function. These findings emphasize the importance of early diagnosis, precise surgical techniques, and individualized treatment planning in improving outcomes for pediatric hepatoblastoma.

## Introduction

Hepatoblastoma, a rare yet critical pediatric liver cancer, accounts for approximately 80% of liver tumors in children, with most cases diagnosed before the age of three. First described in 1898, hepatoblastoma has been identified as a rare but significant intra-abdominal malignancy in pediatric populations [1]. Globally, the incidence of hepatoblastoma is estimated at 1.5-2 cases per million children annually. Prognosis is significantly poorer for individuals diagnosed after the age of five due to its resemblance to hepatocellular carcinoma [2,3]. Historically associated with high mortality rates and poor survival outcomes, advancements in multidisciplinary care have markedly improved prognosis [1].

Hepatoblastoma typically presents as an asymptomatic abdominal mass in the right upper quadrant or epigastric region, often first identified by parents or healthcare providers in infants and toddlers. In some cases, symptoms such as fever, anorexia, weight loss, or fatigue may also be present, although pain and bleeding are relatively rare [1]. Several risk factors have been identified beyond those related to clinical presentation.

Preterm birth and low birth weight significantly increase the risk of hepatoblastoma [4,5]. Neonates with very low birth weight (VLBW) in neonatal intensive care units (NICUs) are particularly vulnerable. Prolonged exposure to oxygen therapy, certain medications, and total parenteral nutrition has been associated with an increased risk of developing hepatoblastoma [1]. These findings underscore the importance of early detection and timely intervention.

Alpha-fetoprotein (AFP), the primary biomarker for hepatoblastoma, is a critical tool for diagnosis and monitoring treatment response [6]. A substantial decline in AFP levels during treatment often signifies a positive response to chemotherapy [7-10]. Abdominal CT imaging is indispensable for determining the PRETEXT stage of hepatoblastoma, which guides surgical planning by assessing tumor extent and resectability. Complete surgical resection is the cornerstone of hepatoblastoma treatment and is crucial for long-term survival [11]. The primary objective of surgery is to achieve complete tumor excision while preserving as much healthy liver tissue as possible. In cases where tumors encase major blood vessels, meticulous surgical dissection is required. Residual microscopic tumors or cancer cells left after surgery significantly increase relapse risk, highlighting the need for margin-free excision [8].

The liver’s remarkable regenerative capacity plays a pivotal role in postoperative recovery. Within six months, the liver typically regains 80-90% of its original volume, while markers like plasma albumin, bilirubin, and prothrombin time show significant improvement within weeks of surgery [10]. Current treatment regimens combine neoadjuvant chemotherapy, surgery, and postsurgical adjuvant chemotherapy. Neoadjuvant chemotherapy reduces the tumor size, facilitating safer surgical resections, while adjuvant chemotherapy targets residual disease and contributes to improved survival rates [9].

Advancements in imaging, surgical techniques, and staging systems have significantly improved hepatoblastoma survival rates [10]. Early diagnosis and well-coordinated treatment planning remain crucial for achieving optimal outcomes [11]. This study examined postsurgical recovery, complication rates, and survival outcomes in children with hepatoblastoma who underwent hepatic resection following neoadjuvant chemotherapy. By evaluating these outcomes, the study provides valuable insights into optimizing multidisciplinary treatment strategies, including surgical techniques and chemotherapy protocols. The aim of this study was to evaluate surgical outcomes in children with hepatoblastoma who underwent hepatic resection after receiving neoadjuvant chemotherapy.

## Materials and methods

This prospective observational study was conducted in the Department of Pediatric Surgery at Bangabandhu Sheikh Mujib Medical University (BSMMU), Dhaka, Bangladesh, from February 2019 to July 2020. During this period, 13 children with hepatoblastoma classified as PRETEXT stages I to III underwent hepatic resection combined with chemotherapy. Informed consent was obtained from the parents or legal guardians of all participants before data collection, and confidentiality was strictly maintained. Ethical approval for the study was granted by the institution’s ethics committee (approval no. BSMMU/2020/251).

Inclusion and exclusion criteria

This study included pediatric patients diagnosed with hepatoblastoma who underwent hepatic resection and chemotherapy. Only patients classified as PRETEXT stages I to III were included, as these stages allow for curative surgical intervention. Patients with PRETEXT stage IV disease or those with distant metastases were excluded to maintain a uniform study population and focus on cases where surgical resection was feasible.

Data collection

A comprehensive medical history was obtained for each patient, followed by thorough clinical examinations. All findings were systematically recorded using a standardized data collection sheet. The diagnosis of hepatoblastoma was confirmed through histopathological examination of biopsy samples. Preoperative imaging, including contrast-enhanced computed tomography (CT) and/or magnetic resonance imaging (MRI), was performed to assess the tumor size, location, vascular involvement, and overall liver anatomy. Additional data collected included liver function test (LFT) values, serum AFP levels, and PRETEXT staging, essential for evaluating tumor burden and liver function before surgery.

Intraoperative findings, operative details, and any complications encountered during the surgical procedure were meticulously documented. Standardized surgical techniques were employed to ensure consistency in hepatic resections. Resected tumor specimens, along with a surrounding rim of normal liver tissue, were preserved in 10% formalin and sent for histopathological analysis to assess resection margin status. Postoperative management protocols were uniformly implemented, with modifications made as needed based on individual patient responses. Discharge criteria were standardized to ensure clinical stability before hospital discharge.

Neoadjuvant chemotherapy protocol

All patients received neoadjuvant chemotherapy as part of the treatment protocol for hepatoblastoma. The chemotherapy regimen primarily consisted of cisplatin and doxorubicin, administered in multiple cycles before surgical intervention. The number of chemotherapy cycles received by each patient was documented. The response to chemotherapy was evaluated through serial AFP measurements and radiological imaging to assess tumor shrinkage and changes in tumor vascularity. These assessments were critical in determining surgical resection's optimal timing and feasibility.

Surgical procedure

Hepatic resection was performed based on the tumor location, response to chemotherapy, and anticipated future liver remnant volume. Intraoperative ultrasound was utilized to delineate tumor margins and assess vascular involvement. Depending on tumor characteristics, anatomic resections such as lobectomy or segmentectomy were preferred for localized disease, while non-anatomic resections were performed when necessary to preserve sufficient liver function. Vascular control techniques, including the Pringle maneuver or selective vascular occlusion, were employed to minimize intraoperative blood loss. Intraoperative frozen section analysis was conducted to confirm negative resection margins and ensure complete tumor removal.

Histopathological assessment

Resected hepatoblastoma specimens and surrounding hepatic tissue were subjected to histopathological analysis to evaluate tumor differentiation, response to chemotherapy, and resection margin status. The resection margins were classified as R0 (negative margins), R1 (microscopic residual disease), or R2 (macroscopic residual disease). Additionally, the degree of tumor necrosis and regression following chemotherapy was assessed, as these findings have prognostic significance in determining disease progression and treatment outcomes.

Postoperative management and adjuvant chemotherapy

Following surgery, patients were managed according to a standardized postoperative care protocol, which closely monitored hepatic function, coagulation parameters, nutritional status, and overall clinical recovery. Pain management and supportive care measures were optimized to enhance postoperative recovery. The need for adjuvant chemotherapy was determined based on histopathological findings, margin status, and tumor stage. Chemotherapy regimens were adjusted according to individual patient requirements, aiming to minimize the risk of recurrence while reducing treatment-related toxicity.

Follow-up protocol

Patients were followed up at one, three, and six months postoperatively to assess hepatic regeneration and monitor for disease recurrence. Preoperative LFTs and serum AFP levels were used as baseline values for comparison with postoperative results. During the first follow-up visit at one month post-surgery, LFTs, AFP levels, and sonographic evaluation of the residual liver volume and echotexture were performed. The same assessments were repeated at three months to monitor continued hepatic recovery. The six-month follow-up focused on evaluating hepatic regeneration through LFTs, AFP measurements, and sonographic imaging to ensure sustained liver function and the absence of recurrent disease. Long-term follow-up beyond six months was conducted to monitor overall survival, liver function, and potential recurrence, ensuring a comprehensive assessment of patient outcomes after hepatic resection and chemotherapy.

Data analysis

Data were collected using a standardized data collection sheet, including demographic information, medical history, PRETEXT staging, clinical findings, and test results. Numerical data were analyzed using percentages, proportions, ratios, means ± SD, and medians. A t-test was used to compare outcomes across different months, with a significance level set at p < 0.05. Statistical analysis was performed using IBM SPSS Statistics for Windows, Version 23 (Released 2015; IBM Corp., Armonk, New York, United States).

## Results

A total of 13 children diagnosed with hepatoblastoma were included in the study. Among the participants, 12 (92.3%) were male, and one (7.7%) was female, with a male-to-female ratio of 12:1. The mean age at diagnosis was 4.44 ± 2.58 years. Most patients, 7 (53.8%), were under three years of age, followed by 5 (38.5%) between 3 and 10 years, and 1 (7.7%) over 10 years of age (Table 1). 

**Table 1:** Demographic characteristics of the study population

Variables	Total number N (%)
Age (in years)	
<3	7 (53.8%)
3-10	5 (38.5%)
>10	1 (7.7%)
Mean±SD	4.44 ± 2.58
Gender	
Male	12 (92.3%)
Female	1 (7.7%)

According to the PRETEXT classification system, seven (53.85%) were categorized as stage III, while five (38.46%) were stage II, and one (7.69%) was stage I. Regarding the type of hepatic resection, eight (61.54%) underwent major resections, four (30.77%) underwent minor resections, and one (7.69%) underwent an extended resection. Histopathological findings revealed fetal-type tumors in six children (46.15%), epithelial-type tumors in five children (38.45%), and mixed tumors in two children (15.4%) (Table 2).

**Table 2:** Clinical characteristics of the study population

Variables	Total number N (%)
PRETEXT category	
I	1 (7.69%)
II	5 (38.46%)
III	7 (53.85%)
Type of hepatic resection	
Extended	1 (7.69%)
Major	8 (61.54%)
Minor	4 (30.77%)
Biopsy of hepatoblastoma	
Epithelial	5 (38.45%)
Fetal	6 (46.15%)
Mixed	2 (15.4%)

The mean preoperative AFP level was 13,251.1 ± 1164.7 ng/mL. These levels significantly decreased post-surgery, with a mean of 112 ± 367 ng/mL at one month, 5.38 ± 3.19 ng/mL at three months, and 3.69 ± 2.19 ng/mL at six months (p < 0.001) (Table 3). 

**Table 3:** Comparison of the AFP level at the preoperative period with postoperative one month, three months, and six months AFP: alpha-fetoprotein; * indicates a significant p-value

AFP (ng/ml)	Preoperative (N=13)	Postoperative (N=13)			Preoperative vs postoperative first month		Preoperative vs postoperative third month		Preoperative vs postoperative sixth month		Postoperative first month vs third month		Postoperative first month vs sixth month		Post three months vs six months			
		1 month Mean ± SD	3rd month Mean ± SD	6th month Mean ± SD	t test value	p-value	t test	p-value	t test	p-value	t test	p-value	t test	p-value		t test	p-value	
Mean ± SD	13251.1 ± 1164.7	112 ± 367	5.38 ± 3.19	3.69 ± 2.19	3.58	0.0015^*^	4.09	0.0004^*^	4.09	<0.001^*^	1.05	0.30	1.06	0.29		1.57	0.12	
Median	694	9.18	5.4	3.2														
Range	66.4 to 3020	3.45 to 1333.8	1.83 to 14.22	1.53 to 7.20														

LFTs also demonstrated significant improvements. ALT levels decreased from 34.38 ± 8.37 U/L preoperatively to 26.38 ± 8.60 U/L at six months (p = 0.02), while AST levels dropped from 44.92 ± 10.20 U/L to 33.23 ± 7.65 U/L (p = 0.003). Other parameters, such as prothrombin time (PT) (12.23 ± 0.45 seconds preoperatively to 12.43 ± 0.92 seconds at six months) and international normalized ratio (INR) (1.03 ± 0.05 preoperatively to 1.04 ± 0.08 at six months), remained stable throughout the follow-up period, indicating no adverse impact on coagulation profiles (Table 4). 

**Table 4:** Comparison of the liver function test at preoperative period with postoperative one month, three months, and six months PT: prothrombin time (seconds); APTT: activated partial thromboplastin time (seconds); INR: international normalized ratio; SGPT: serum glutamic-pyruvic transaminase (units/liter); SGOT: serum glutamic-oxaloacetic transaminase (units/liter); S.T.P: serum total protein (grams/liter); S.Alb: serum albumin (grams/liter); S.Alb:S.Glb: serum albumin-to-serum globulin ratio; S.Bil (t): serum bilirubin total (milligrams/deciliter); S.Bil (d): serum bilirubin direct (milligrams/deciliter); S.ALP: serum alkaline phosphatase (units/liter); GGT: gamma-glutamyl transferase (units/liter) * indicates a significant p-value

Variables	Preoperative (N= 13)	Postoperative outcomes			Preoperative vs first month		Preoperative vs third month		Preoperative vs sixth month		Post first month vs third month		Post one month vs sixth month		Post three months vs sixth month	
		1 month	3 months	6 months												
	Mean ± SD	Mean ± SD	Mean ± SD	Mean ± SD	t-test	p-value	t test	p-value	t test	p-value	t test	p-value	t test	p-value	t test	p-value
PT (sec)	12.23 ± 0.45	13.78 ± 5.61	12.08 ± 0.43	12.43 ± 0.92	-0.99	0.33	0.87	0.39	-0.7	0.48	1.09	0.28	0.86	0.40	-1.24	0.22
APTT (sec)	32.36 ± 3.11	30.03 ± 6.91	29.32 ± 2.01	31.53 ± 3.56	1.11	0.27	2.96	0.006^*^	-0.63	0.53	0.36	0.72	-0.7	0.49	-1.96	0.06
INR	1.03 ± 0.05	1.02 ± 0.08	1.02 ± 0.04	1.04 ± 0.08	0.38	0.70	0.56	0.57	-0.38	0.70	0	1.00	-0.64	0.52	-0.81	0.42
SGPT (U/L)	34.38 ± 8.37	34.62 ± 12.00	35.69 ± 8.96	26.38 ± 8.60	-0.06	0.95	-0.39	0.70	2.4	0.02^*^	-0.26	0.79	2.01	0.05	2.7	0.01^*^
SGOT (U/L)	44.92 ± 10.20	43.77 ± 14.93	42.46 ± 12.75	33.23 ± 7.65	0.23	0.82	0.54	0.59	3.31	0.003^*^	0.24	0.811	2.27	0.03^*^	2.24	0.03^*^
S.T.P (gm/l)	66.69 ± 10.42	66.41 ± 18.87	75.15 ± 4.81	67.38 ± 8.05	0.05	0.96	-2.66	0.01^*^	-0.19	0.85	-1.62	0.11	-0.17	0.86	2.99	0.006^*^
S.Alb (gm/l)	41.85 ± 6.12	39.34 ± 11.19	45.15 ± 3.95	40.92 ± 4.25	0.71	0.48	-1.63	0.11	0.45	0.65	-1.77	0.09	-0.48	0.63	2.63	0.01^*^
S.Alb:S.Glb	1.34 ± 0.17	1.34 ± 0.31	1.49 ± 0.22	1.42 ± 0.25	0	1.00	-1.95	0.06	-0.95	0.34	-1.42	0.16	-0.72	0.47	0.76	0.45
S.Bil (T) mg/dl	0.39 ± 0.10	0.52±0.24	0.41 ± 0.15	0.37 ± 0.20	-1.8	0.08	-0.4	0.69	0.32	0.74	1.4	0.17	1.73	0.09	0.58	0.56
S.Bil (D) mg/dl	0.11 ± 0.07	0.12 ± 0.19	0.05 ± 0.02	0.07 ± 0.06	-0.18	0.86	2.97	0.006^*^	1.56	0.143	1.32	0.19	0.9	0.37	-1.14	0.26
S.ALP (U/L)	222.1 ± 46.7	193.8 ± 75.8	139.6 ± 26.3	135.2 ± 37.7	1.15	0.26	5.55	0^*^	5.22	0.00^*^	2.44	0.02^*^	2.5	0.019^*^	0.35	0.73
GGT (U/L)	52.62 ± 20.85	46.15 ± 27.36	26.62 ± 10.51	26.69 ± 15.36	0.68	0.50	4.01	0.0005^*^	3.61	0.0014^*^	2.4	0.02^*^	2.2	0.03^*^	-0.01	0.98

Notably, all 13 (100%) participants exhibited homogeneous hepatic echotexture at one, three, and six months post-surgery, and no local recurrences were observed, underscoring the success of the surgical and adjuvant treatment approach (Table 5).

**Table 5:** Postoperative hepatic echotexture and local recurrences at different time periods after operation

Variables	One month N (%)	Three months N (%)	Six months N (%)
Homogenous			
Yes	13 (100.0%)	13 (100.0%)	13 (100.0%)
No	0 (0.0%)	0 (0.0%)	0 (0.0%)
Local recurrence			
Yes	0 (0.0%)	0 (0.0%)	0 (0.0%)
No	13 (100.0%)	13 (100.0%)	13 (100.0%)

Hepatic regeneration was observed throughout follow-up, with the mean residual liver volume increasing from 259.4 ± 71.04 cm³ at one month to 329.3 ± 73.22 cm³ at six months. The highest regeneration rate was observed during the first six months postoperatively, with an average monthly regeneration of 13.97 ± 2.87 cm³ (p < 0.001) (Table 6). 

**Table 6:** Comparison of residual liver volume at postoperative one month, three months, and six months period p-value less than 0.05 was considered significant.

Residual liver volume (cc)	One month	Three months	Six months	Postoperative one month vs postoperative three months		Postoperative one month vs postoperative six months		Postoperative three months vs postoperative six months	
	(n=13)	(n=13)	(n=13)	t test	p-value	t test	p-value	t test	p-value
Mean ± SD	259.4 ± 71.04	292.0 ± 85.07	329.3 ± 73.22	1.49	<0.001	3.49	<0.001	1.69	<0.001

All 13 (100%) participants exhibited favorable hepatic regeneration, with none showing delayed or absent regeneration (Table 7).

**Table 7:** Postoperative hepatic regeneration (volume) rate (cc) per month

Regeneration rate (per month)	Total number N (%)
Yes	13 (100%)
No	0 (0%)
Mean±SD	13.97 ± 2.87
Min-Max (CC)	9.8 to 18.52

## Discussion

Hepatoblastoma is the most common liver tumor in children, accounting for approximately 80% of malignant liver tumors. Over the past two decades, the incidence of hepatoblastoma has increased from 0.6 to 1.2 cases per million individuals, with an annual rate ranging from 0.8 to 1.5 cases per million [12]. This rise is primarily attributed to the increasing number of premature births and infants with VLBW [13]. These findings emphasize the importance of addressing risk factors, including preterm birth and low birth weight, in hepatoblastoma development.

The gender distribution in our study group (92.3% male) aligns with prior research, which consistently shows a higher prevalence of hepatoblastoma in men than in women [14]. Most patients (53.8%) were younger than three years, which reflects the typical age of presentation for hepatoblastoma in early childhood [15]. These observations corroborate findings by Li et al. [16], who reported that hepatoblastoma primarily affects children aged 0-3 years, with a male-to-female ratio of approximately 2:1.

Regarding clinical characteristics, most patients in this study were classified as PRETEXT category III (53.85%), indicating a significant extent of disease at diagnosis. This finding is consistent with data from the International Childhood Liver Tumors Strategy Group (SIOPEL), which has reported that many patients present with advanced disease requiring neoadjuvant chemotherapy before surgery [16,17]. Neoadjuvant therapy not only reduces the tumor size but also helps eliminate microscopic metastatic foci, thereby facilitating safer surgical resections [6].

Major hepatic resections, which involve the removal of a substantial portion of the liver, were the most commonly performed procedures in this study, accounting for 61.54% of cases. This aligns with the findings of Murawski et al. [17], who emphasized the importance of complete tumor resection for long-term survival in hepatoblastoma. Among the histological subtypes observed, 46.15% of tumors were fetal-type hepatoblastomas, which are associated with a more favorable prognosis compared to other variants [15].

Postoperative changes in LFTs can have significant physiological implications that may influence patient outcomes. The liver plays a critical role in metabolism, detoxification, and protein synthesis, and any disturbances in liver function can affect recovery and overall prognosis [18]. In this study, liver enzyme levels, including alanine aminotransferase (ALT) and aspartate aminotransferase (AST), remained within a normal range or returned to baseline shortly after surgery, suggesting that hepatic regeneration occurred efficiently. This rapid restoration of liver function is crucial, as persistent elevation of liver enzymes or impaired synthetic function, reflected by low albumin or prolonged prothrombin time, could indicate ongoing hepatic stress or inadequate regeneration [19]. Furthermore, bilirubin levels remained stable, suggesting that bile excretion was not significantly impaired, which is essential for preventing complications such as cholestasis and hepatic dysfunction [20].

Physiological changes due to postoperative liver function fluctuations can also impact long-term outcomes, particularly in pediatric patients. The ability of the liver to regenerate is well-documented. Still, factors such as nutritional status, post-surgical inflammatory responses, and underlying liver conditions can influence the speed and quality of recovery [21]. Inadequate hepatic regeneration or prolonged liver dysfunction can lead to complications such as coagulopathy, metabolic imbalances, and increased susceptibility to infections, all of which could negatively impact survival and quality of life. Conversely, this study's observed rapid stabilization of liver function parameters supports the feasibility and safety of aggressive surgical management in hepatoblastoma patients. These findings align with previous research, indicating that most biochemical liver markers normalize within weeks after resection, reinforcing the liver’s remarkable regenerative capacity [22].

Postoperative AFP levels significantly declined, demonstrating the effectiveness of surgical intervention. Specifically, AFP levels dropped from a mean preoperative level of 13,251.1 ng/mL to 3.69 ng/mL at six months. This outcome highlights the role of AFP as a reliable biomarker for assessing treatment response and monitoring recurrence, as demonstrated by Tomlinson et al. [23]. Regular AFP monitoring and liver ultrasonography during the first five years post-surgery remain critical for the early detection of recurrence.

In addition to favorable surgical outcomes, preserving liver function plays a crucial role in recovery. No significant differences were observed in LFT parameters, including ALT, AST, and bilirubin levels, among patients undergoing major, minor, or extended hepatic resections. These findings align with that of Needham et al. [10], who reported that most biochemical liver function markers normalize within 1-2 weeks after significant liver resections in children. Full biochemical recovery, including normalization of albumin and bilirubin levels, is typically observed within 3-6 weeks.

The absence of local recurrences and consistent hepatic echotexture throughout the six-month follow-up highlights the success of surgical interventions in this cohort. While local recurrence remains a significant concern in hepatoblastoma treatment, reported in up to 30% of cases without appropriate management [24,25], this study demonstrates the importance of achieving margin-free excision and precise surgical techniques to mitigate this risk.

Significant hepatic regeneration was observed, with mean liver volumes increasing from 259.4 cm³ at one month to 329.3 cm³ at six months postoperatively. The highest regeneration rates were observed during the first six months, with a mean regeneration rate of 13.97 cm³ per month. Similar findings were reported by Nagino et al. [26], who noted that liver volume increases rapidly during the first few weeks post-surgery and stabilizes at 74-83% of its original size within 12 months. These results underscore the remarkable regenerative capacity of the liver and its critical role in ensuring long-term recovery and functionality.

Strength

This study had several strengths that enhanced its clinical significance and contribution to hepatoblastoma research. It provided a comprehensive analysis of patient demographics, tumor classification, surgical interventions, and postoperative outcomes, allowing for a clearer understanding of disease progression and treatment response. A key strength was the AFP level assessment, which showed a significant decline after surgery, reinforcing AFP’s reliability as a biomarker for treatment monitoring and recurrence detection. Additionally, the study demonstrated the liver’s strong regenerative capacity, with significant increases in liver volume post-surgery, confirming the safety and feasibility of major hepatic resections. The stability of liver function tests further supported the effectiveness of surgical treatment without compromising essential liver functions. Another major strength was the absence of local recurrences during the six-month follow-up, suggesting that complete tumor removal and precise surgical techniques played a crucial role in minimizing relapse risk. These findings aligned with international treatment guidelines, such as those from the International Childhood Liver Tumors Strategy Group (SIOPEL), adding to the study’s credibility. Furthermore, the study reinforced the importance of neoadjuvant chemotherapy, complete tumor resection, and AFP monitoring as essential strategies in hepatoblastoma management. By highlighting these key aspects, the study contributed to evidence-based clinical decision-making and provided valuable insights for improving future treatment approaches in pediatric liver tumors.

Limitations

While this study provides valuable insights, its small sample size and short follow-up period limit its generalizability. A larger cohort and extended follow-up would provide more robust data on long-term survival rates and recurrence patterns. Exploring other prognostic factors, such as genetic mutations and responses to specific chemotherapy regimens, could provide valuable insights into improving hepatoblastoma treatment strategies.

## Conclusions

This study highlights the efficacy of integrating hepatic resection with neoadjuvant chemotherapy in treating pediatric hepatoblastoma. The significant decline in AFP levels following surgery underscores the effectiveness of tumor resection in achieving disease control. AFP also serves as a reliable biomarker for monitoring treatment response. 

Improvements in LFTs and substantial hepatic regeneration within six months further demonstrate the liver’s remarkable capacity for recovery, ensuring the restoration of its metabolic and physiological functions. These findings emphasize the importance of early diagnosis, precise surgical techniques, and a multidisciplinary approach to achieving favorable outcomes. This study contributes to the growing body of evidence supporting the efficacy of combined treatment strategies in hepatoblastoma management. Future research should focus on larger cohorts and extended follow-up periods to better understand long-term outcomes, recurrence patterns, and the impact of genetic factors and responses to chemotherapy on treatment success.
